# Supplementary material for: Characterization of the Basal and mTOR-Dependent Acute Pulmonary and Systemic Immune Response in a Murine Model of Combined Burn and Inhalation Injury
Source: Int J Mol Sci. 2022 Aug 7;23(15):8779. doi: 10.3390/ijms23158779 (PMC9368856; doi:10.3390/ijms23158779)
Supplement: Supplementary file 1 [file ijms-23-08779-s001.zip › ijms-1821115-supplementary.pdf]

Lung

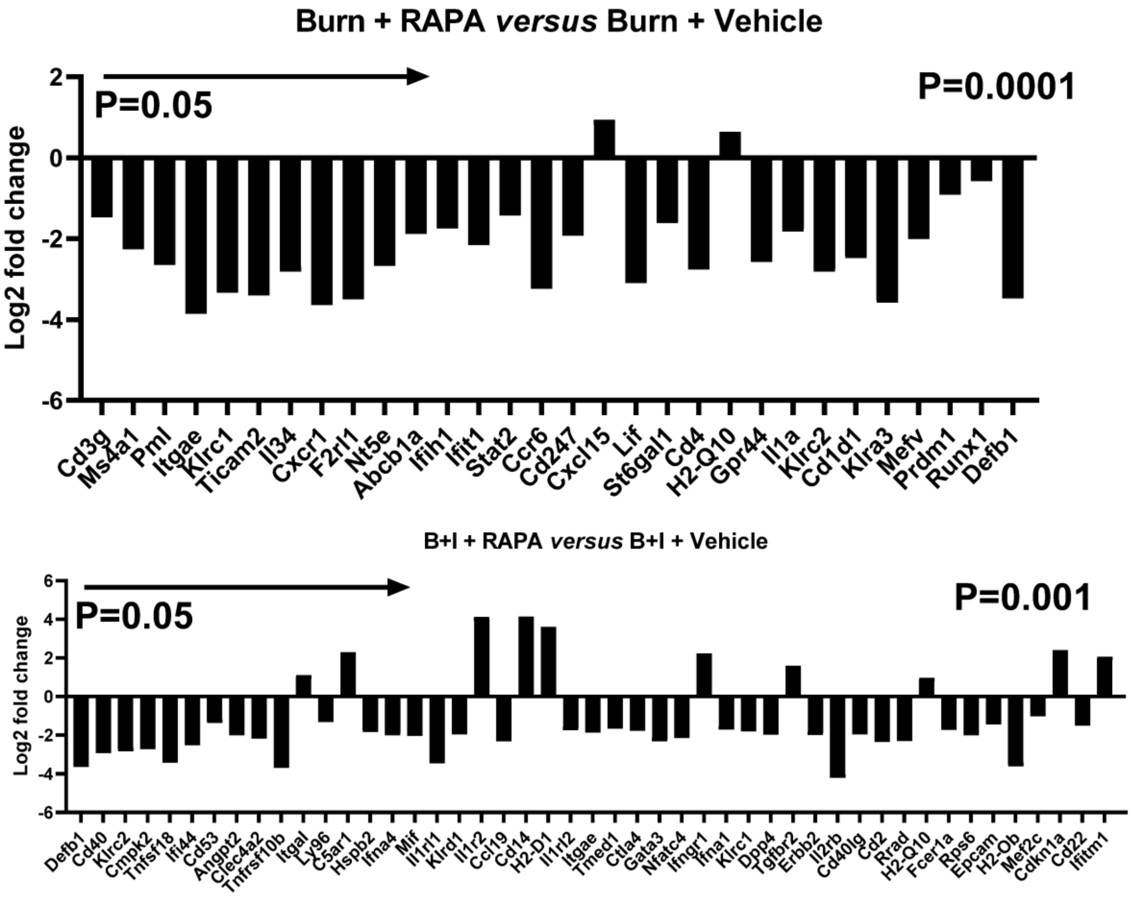

Figure S1: Significantly altered genes (p<0.05) of burn + Rapamycin versus vehicle control and B+I + Rapamycin versus B+I vehicle control in the lung.

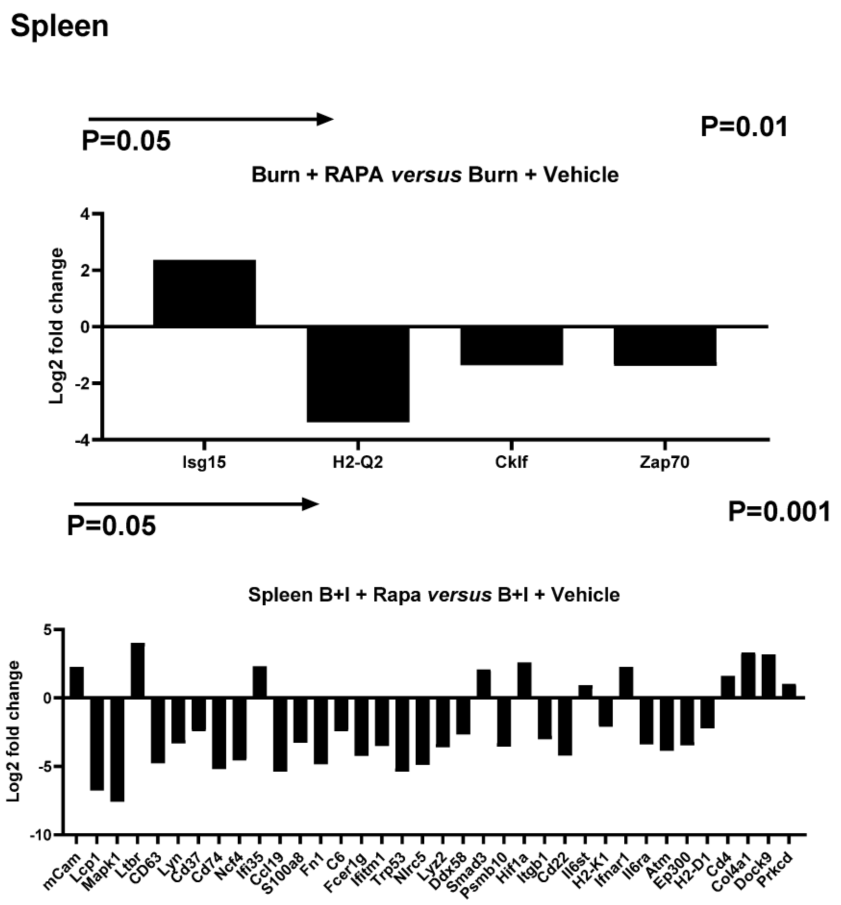

Figure S2: Significantly altered genes ($p<0.05$) of burn + Rapamycin versus vehicle control and B+I + Rapamycin versus B+I vehicle control in the spleen.
